# Association between tocilizumab treatment and clinical outcomes of COVID-19 patients: a systematic review and meta-analysis

**DOI:** 10.18632/aging.203834

**Published:** 2022-01-17

**Authors:** Jingwen Peng, Xiaodong She, Huan Mei, Hailin Zheng, Meihua Fu, Guanzhao Liang, Qiong Wang, Weida Liu

**Affiliations:** 1 Department of Medical Mycology, Institute of Dermatology, Chinese Academy of Medical Science and Peking Union Medical College, Nanjing 210042, Jiangsu, People’s Republic of China; 2 Center for Global Health, School of Public Health, Nanjing Medical University, Nanjing 211166, Jiangsu, People’s Republic of China; 3 Jiangsu Key Laboratory of Molecular Biology for Skin Diseases and STIs, Nanjing 210042, Jiangsu, People’s Republic of China

**Keywords:** tocilizumab, COVID-19, efficacy, meta-analysis, randomized controlled trial

## Abstract

To explore and summarize the association between treatment with tocilizumab and clinical outcomes in COVID-19 patients. We performed a systematic review and meta-analysis (10 RCTs including 3378 patients in the tocilizumab group and 3142 patients in the control group). We systematically searched PubMed and MedRxiv for all RCTs as of June 1, 2021, to assess the benefits and harms of tocilizumab to treat patients with COVID-19. All analyses were carried out using RevMan version 5.4.1. There were nine RCTs published in peer-reviewed journals and one RCTs published as a preprint. The summary RR for all-cause mortality with tocilizumab was 0.89 (95% CI= 0.82-0.96, *P*= 0.003). There was no significant between-trial heterogeneity (*I*^2^= 28%, *P*= 0.19). However, all peer-reviewed RCTs showed no significant associations between treatment with tocilizumab and reductions in all-cause mortality. We notably found that tocilizumab significantly reduced the rate of intubation or death in patients with COVID-19 with 3 RCTs. Across the 8 RCTs, the summary RR for discharge with tocilizumab was 1.10 (95% CI= 1.03-1.16, *P*< 0.00001). There was no significant association of tocilizumab with harm on other patient-relevant clinical outcomes, including increasing secondary infection risk, patients of adverse events, or patients of serious adverse events. Tocilizumab significantly increased the rate of hospital discharges in COVID-19 patients. Still, it did not decrease all-cause mortality or increase the risk of secondary infections, patients of adverse events, or patients for serious adverse events. Evidence that tocilizumab affects clinical outcomes in patients with COVID-19 requires further proof.

## INTRODUCTION

Severe acute respiratory syndrome coronavirus 2 (SARS-CoV-2), a novel human pathogen, is one of the most considerable global challenges facing public health and humanity [1–3]. With the development of the coronavirus disease 2019 (COVID-19) pandemic, there has been unwarranted enthusiasm for using tocilizumab [4–12], but the clinical evidence of its benefits or harm is limited.

COVID-19 is associated with dysregulated immune responses and hyper inflammation, including releasing of proinflammatory cytokines and chemokines. It can cause or worsen acute respiratory distress syndrome and multiple organ failure [13–15]. Several scholars have recently suggested that tocilizumab may be positively associated with a lower risk of intubation or death in patients with severe and critically ill COVID-19 pneumonia [11, 13–20]. Inhibitors of Interleukin 6 (IL-6) or its receptor have successfully treated different cytokine storm syndromes or powerful chimerical antigen receptor T cell -mediated cytokine release syndrome [2, 13]. The randomized evaluation of the COVID-19 therapy platform (RECOVERY) trial is by far the largest randomized clinical trial (RCT) on COVID-19 treatments [21]. It has provided essential evidence for several promising therapies, including hydroxychloroquine, dexamethasone, lopinavir-ritonavir, convalescent plasma, and azithromycin.

Given the previously reported RCTs, we conducted the systematic review and meta-analysis (10 RCTs including 3378 patients in the tocilizumab group and 3142 in the control group) to explore and summarize the association between tocilizumab treatment and clinical outcomes in COVID-19 patients.

## RESULTS

We noted 39 records in the related databases, registries, and other sources. We included 9 RCTs published in peer-reviewed journals and 1 RCTs published as preprints. Of the ten included RCTs, three were in the USA, two were in India, and one each in France, Italy, Brazil, International, and the UK. Only 1 RCT was prematurely interrupted after an interim analysis for futility (NCT04346355). There were three double-blind RCTs (NCT04356937, NCT04372186, and NCT04320615), whereas the other 7 were open-label RCTs (CTRI/2020/05/024959, NCT04331808, NCT04346355, NCT04403685, CTRI/2020/05/025369, NCT02735707, and NCT04381936).

From 9 RCTs published in peer-reviewed journals, there were 2404 patients (1048 to placebo together with the standard of care or only standard of care and 1356 randomized to tocilizumab) in our meta-analysis. There were 4116 patients (2094 to the only standard of care and 2022 randomized tocilizumab) in the RECOVERY trial (NCT04381936). Comorbidities at randomization were universal when reported in most studies. Detailed information on patient characteristics was accessible to all RCTs (Table 1 and Supplementary Table 1).

**Table 1:** Characteristics of the 8 RCTs in the meta-analysis.

**Author**		**Olivier Hermine**	**Carlo Salvarani**	**J.H. Stone**	**Carlos Salama**	**I.O. Rosas**	**Viviane C Veiga**	**Suresh Kumar**	**Arvinder S Soin**	**Anthony C. Gordon**	**Peter W Horby**
Trial registration		NCT 04331808	NCT 04346355	NCT 04356937	NCT 04372186	NCT 04320615	NCT 04403685	CTRI/2020/05/024959	CTRI/2020/05/025369	NCT 02735707	NCT 04381936
Time		20201020	20201020	20201021	20201217	20210225	20210120	20201201	20210504	20210422	20210211
Country		France	Italy	USA	USA	USA	Brazil	India	India	International	UK
Race		Caucasian	Caucasian	Mix	Mix	Mix	Mix	Asian	Caucasian	Mix	Mix
Disease severity		Moderate or severe	NA	Severe	NA	Severe	Severe or critical	Moderate or severe	Moderate or severe	Critical	Severe
Dose description		8mg/kg maximum 800 mg	8mg/kg maximum 800 mg	8mg/kg maximum 800 mg	8mg/kg maximum 800 mg	8mg/kg maximum 800 mg	8mg/kg maximum 800 mg	1.6mg/kg and continued with 0.8 mg/kg dose weekly regimen	6mg/kg maximum 800 mg	8mg/kg maximum 800 mg	400mg-800mg
Type of control		Usual care	Standard care	Placebo plus standard care	Placebo plus standard care	Placebo plus standard care	Standard care	Best supportive care	Standard care	Standard care	Standard care
Study type		Open-label RCT	Open-label RCT	Double-blind RCT	Double-blind RCT	Double-blind RCT	Open-label RCT	Open-label RCT	Open-label RCT	Open-label RCT	Open-label RCT
Peer-reviewed		Yes	Yes	Yes	Yes	Yes	Yes	Yes	Yes	Yes	No
Publication format		Publish	Publish	Publish	Publish	Publish	Publish	Publish	Publish	Publish	Preprint
No. planned of inclusion		131	126	1560	445	479	129	36	183	2046	21550
No. included		130	123	243	377	438	129	30	180	865	4116
Tocilizumab		63	60	161	249	294	65	20	91	353	2022
Control		67	63	82	128	144	64	10	88	402	2094
Mortality	Tocilizumab	28d:7	30d:2	28d: 9	28d:26	28d:58	28d:14	30d:0	28d:11	30d:87	28d:596
	Control	28d:8	30d:1	28d:3	28d:11	28d:28	28d:6	30d:3	28d:15	30d:134	28d:694
Discharge	Tocilizumab	28d:52	30d:54	28d:147		28d:180	28d:35	30d:16		30d:190	28d:1093
	Control	28d:49	30d:58	28d:72		28d:74	28d:31	30d:6		30d:184	28d:999
Patients of adverse events	Tocilizumab	28d:28			60d:127	28d:228	28d:29	30d:18	28d:30		
	Control	28d:36			60d:67	28d:116	28d:21	30d:4	28d:22		
Patients of serious adverse events	Tocilizumab	28d:20		28d:28		28d:103	28d:11		28d:15	30d:9	
	Control	28d:29		28d:12		28d:55	28d:7		28d:15	30d:11	
Secondary infection	Tocilizumab	28d:2	30d:1	28d:13	60d:25	28d:113	28d:10	30d:1	28d:5	30d:1	
	Control	28d:14	30d:4	28d:14	60d:16	28d:58	28d:10	30d:3	28d:5	30d:0	
Incubation or death	Tocilizumab	15		17							571(1754)
	Control	24		10							687(1800)

The mix of Severity, Symptoms of the disease include moderate, severe, and critical; Mix of Race, including Asian, Caucasian, African, and so on; RCT, Randomized controlled trial; NA, no appearance.

### Association of tocilizumab with clinical outcomes

For all RCTs, the all-cause mortality in patients receiving tocilizumab was 23.98% (810/3378) and 28.74% (903/3142) in control patients. We found the summary RR for all-cause mortality with tocilizumab was 0.89 (95% CI= 0.82-0.96, *P*= 0.003). There was no significant between-trial heterogeneity (*I*^2^= 28%, *P*= 0.19). However, 9 peer-reviewed RCTs showed that no significant association between tocilizumab treatment and all-cause mortality reduction (RR= 0.87, 95% CI= 0.73-1.04, *P* = 0.13).

**Figure 1:**
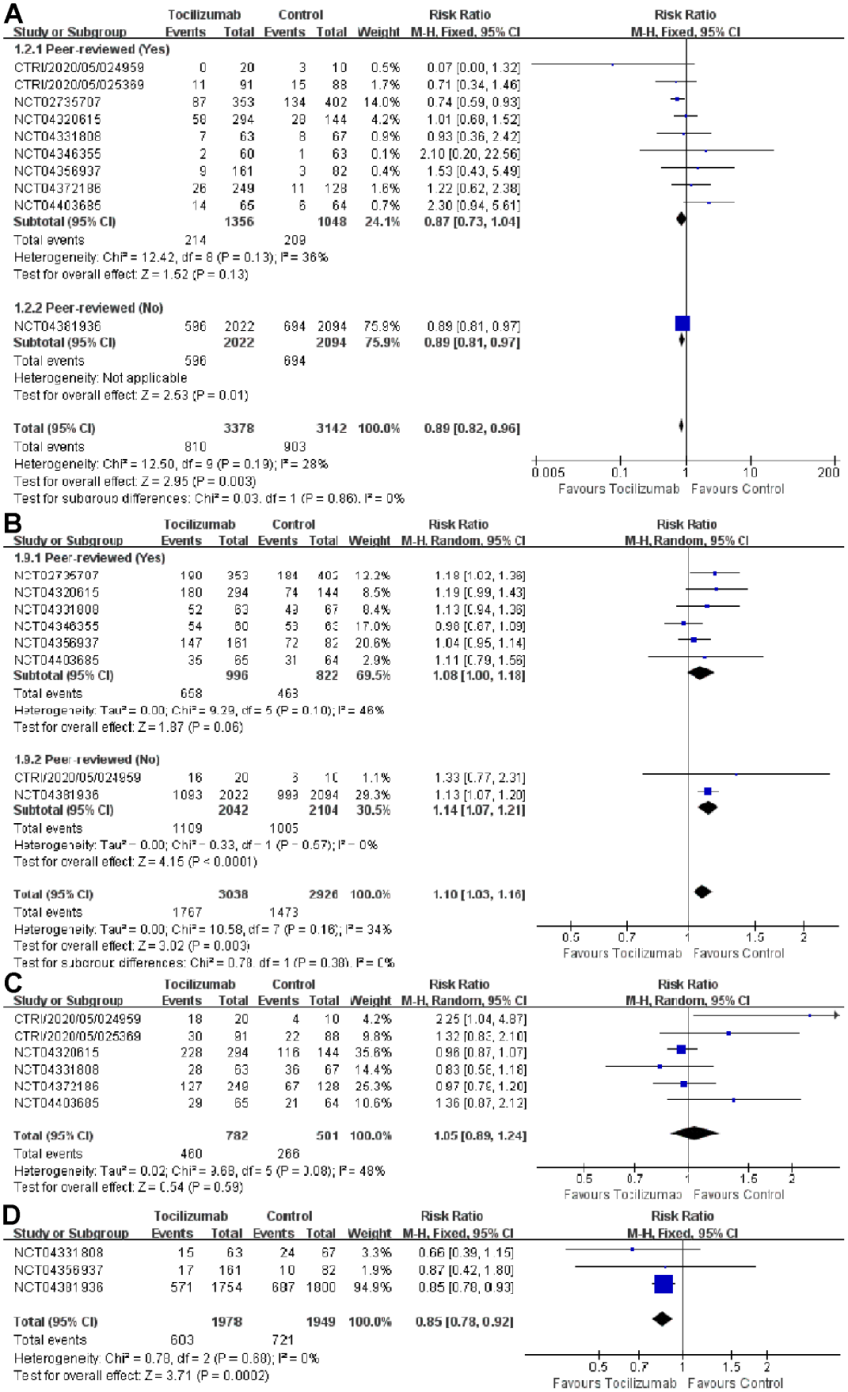
Association of tocilizumab with all-cause mortality, discharge, patients of adverse events, and date of incubation or death in the published study and preprint study (**A**) all-cause mortality (**B**) discharge (**C**) patients of adverse events (**D**) date of incubation or death.

Three double-blind RCTs received placebo, no significant association between tocilizumab treatment and all-cause mortality reduction (RR= 1.10, 95% CI= 0.79-1.54, *P*= 0.57). We notably found that tocilizumab significantly reduced the rate of intubation or death in patients with COVID-19 with 3 RCTs (RR= 0.85, 95% CI= 0.78-0.92, *P*= 0.0002) (Figure 1A, 1D, and Supplementary Figure 1).

We conducted a Begg/Egger test and used a funnel plot to assess the publication bias of our meta-analysis (*P*-value of publication bias was 0.596). We also performed a sensitivity analysis by omitting one study when calculating the summary results. After eliminating the RECOVERY trial (NCT04381936), the combined OR value and 95%CI changed from positive to adverse. As the amount of the RECOVERY trial data accounts for 76% of the total data and the risk of bias in the RECOVERY trial was considered high, which may cause the combined results of the RECOVERY trial to be not very reliable (Supplementary Figure 2).

Across the 8 RCTs, the summary RR for discharge with tocilizumab was 1.10 (95% CI=1.03-1.16, *P*<0.0001). Similar results were also observed for the preprint, peer-reviewed RCTs, double-blind (placebo plus standard care) and open label RCTs (standard care) for discharge (RR= 1.08, 95% CI= 1.00-1.18, *P*=0.06; RR= 1.14, 95% CI= 1.07-1.21, *P*<0.0001; RR= 1.10, 95% CI= 0.93-1.29, *P*=0.27; RR= 1.10, 95% CI= 1.03-1.18, *P*=0.008) (Figure 1B and Supplementary Figure 3).

We unobserved a significant association between tocilizumab and a decreased risk of secondary infections in the overall analysis (RR= 1.05, 95% CI= 0.89-1.24). However, there was a slight between-trial heterogeneity (*I*^2^= 48%; *P*= 0.08). We did not discover significant associations between tocilizumab treatment and secondary infection risk in peer-reviewed, preprint RCTs, double-blind (placebo plus standard care), and open-label RCTs (standard care) subgroups. We also failed to find significant associations between tocilizumab and patients of adverse events as well as patients of serious adverse events (Figure 1C and Supplementary Figures 4, 5).

### Risk of bias

**Figure 2:**
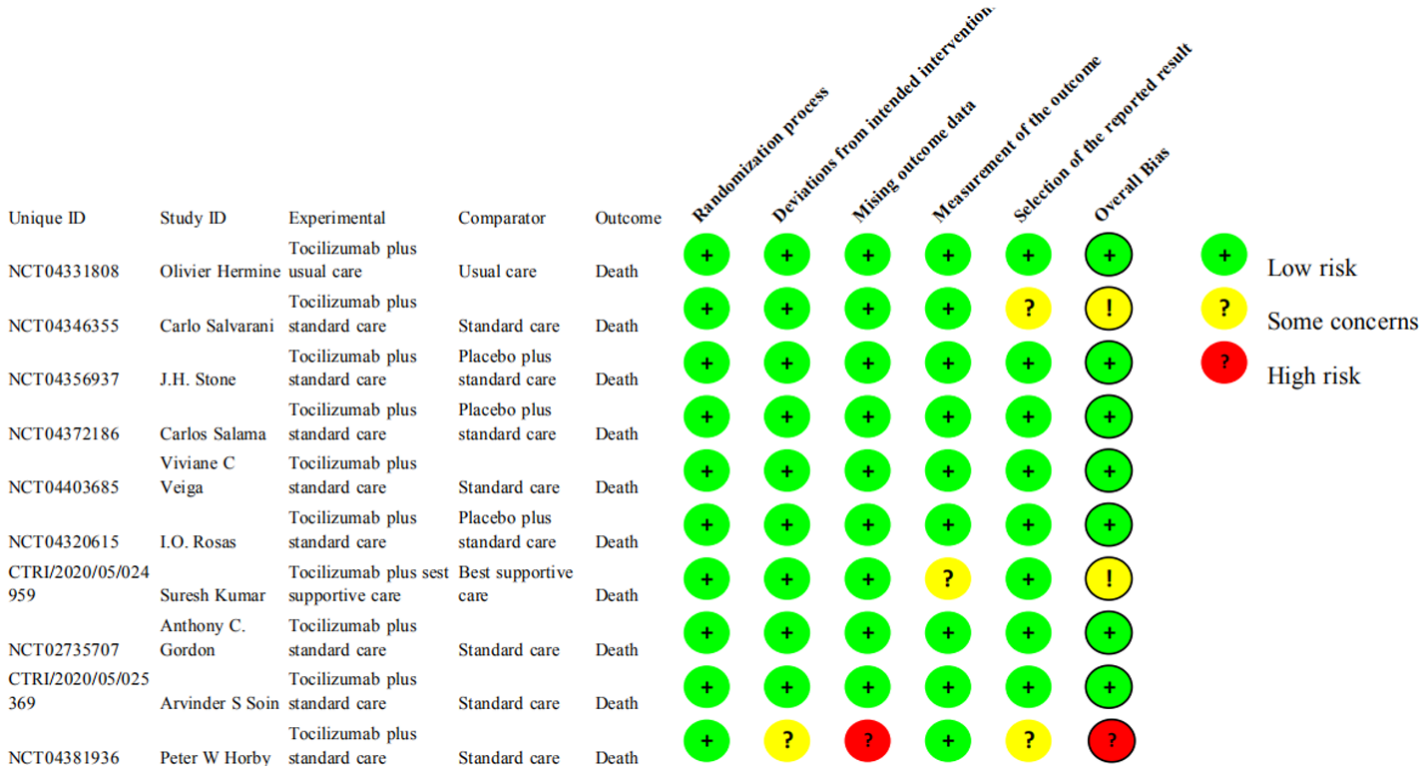
Risk of bias assessments for the outcomes of all RCTs.

The risk of bias for all-cause mortality, in-patient discharge rate, number of patients experiencing serious adverse events and adverse events, number of patients’ intubation or death, and number of secondary infections were thought low for 8 of the 10 RCTs. The other two RCTs have some concerns (NCT04346355), and 1 RCT was considered high (NCT04381936) (Figure 2).

## DISCUSSION

9 RCTs were published in peer-reviewed journals in the meta-analysis. We revealed that tocilizumab treatment was not significantly associated with reducing all-cause mortality among COVID-19 patients compared with placebo plus standard of care or standard of care alone. However, combined with additional 1 RCTs published in preprint journals, we observed that tocilizumab treatment significantly reduced all-cause mortality in COVID-19 patients. The risk of bias in the RECOVERY trial (NCT04381936) is very high, and the combined results of the RECOVERY trial are not very reliable. Evidence that tocilizumab reduces all-cause mortality in COVID-19 patients requires further proof.

In the overall analysis, we discovered a significant increase in hospital discharge rates after patients with COVID-19 pneumonia received tocilizumab. We observed similar results for the peer-reviewed, preprint RCTs, double-blind (placebo plus standard care), and open-label RCTs (standard care) for discharge. We notably also found that tocilizumab significantly reduced the rate of intubation or death in COVID-19 patients in 3 RCTs.

Tocilizumab was not significantly associated with harm on other patient-relevant clinical outcomes, including increasing secondary infection risk, patients of adverse events, or patients of serious adverse events. The possible reason is tocilizumab treatment significantly increased COVID-19 discharge rates in patients with mild disease compared to standard care alone or placebo. In severe or critically ill patients, because mortality is a multifactorial outcome. In critically ill patients, medical personnel use all available medical means to save patients' lives; we did not find that tocilizumab significantly reduced all-cause mortality.

We found our evidence was dominated mainly by the RECOVERY trial (NCT04381936), which amounted to 76% of the meta-analysis weight [21]. After eliminating the RECOVERY trial, we failed to find a significant association between tocilizumab and all-cause mortality, intubation, or mortality in patients with COVID-19 [21]. However, excluding the RECOVERY trial, we found that tocilizumab effectively increased patients’ discharge rate with COVID-19.

Carlo Salvarani et al. [7] was prematurely interrupted the trial after an interim analysis for futility. Three randomized, double-blind, placebo-controlled trials reported tocilizumab treatment did not significantly result in better clinical status or lower mortality than placebo at point time [5, 6, 8]. Three open-label published RCTs also said tocilizumab treatment plus standard care was not slightly superior to usual care alone in improving clinical outcomes [4, 7, 9].

Viviane C Veiga et al. [9] reported that two patients in the standard care group received tocilizumab treatment. Peter W Horby et al. [21] also said that forty-four participants (3%) assigned to usual care received at least one dose of tocilizumab in the RECOVERY trial. Timotius Ivan Hariyanto et al. [22] also observed that tocilizumab is effective in reducing the biomarkers of the COVID-19 infection. Overall, we believe that tocilizumab significantly increased the discharge rate of patients with COVID-19 but did not decrease all-cause mortality and increase the risk of secondary infection, patients with adverse events, or patients with serious adverse events in the meta-analysis. Therefore, we recommend that clinicians be cautious in using tocilizumab in patients with COVID-19pneumonia.

We have to consider several limitations in our study. Firstly, in the absence of every patient’s clinical test data, patients with high clinical indicators (such as IL-6, C-reactive protein, and so on) will benefit most when tocilizumab treatment. Secondly, two of the 10 RCTs had some concerns, and one RCT have an increased risk of bias. The RECOVERY trial (NCT04381936) accounted for 76% of the weight in our meta-analysis. Thirdly, two RCTs reported that some patients in the standard care group also received tocilizumab, which may affect the efficacy evaluation [1, 23–25].

## CONCLUSIONS

Tocilizumab significantly increased the discharge rate of patients with COVID-19. Still, it did not decrease all-cause mortality and increased the risk of secondary infection, patients of adverse events, or patients of serious adverse events.

## MATERIALS AND METHODS

**Figure 3:**
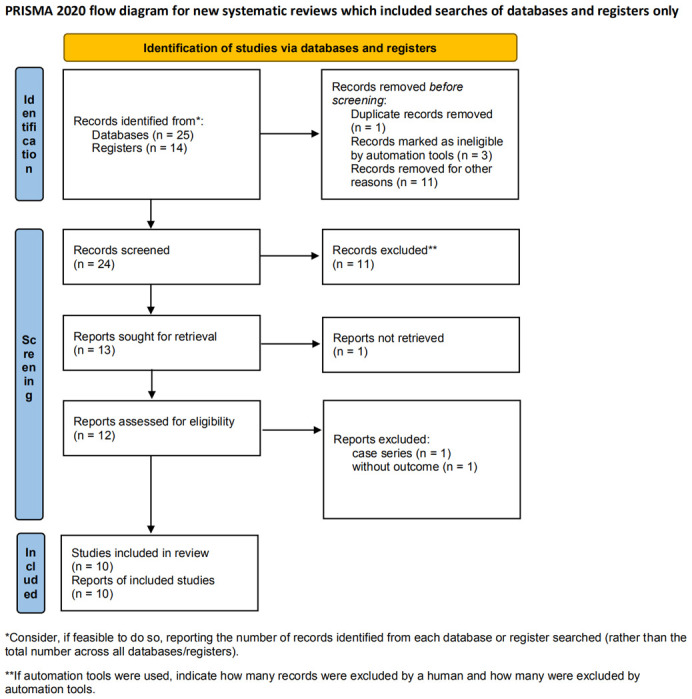
Flow diagram of the study selection process.

We conducted the systematic review and meta-analysis of 10 RCTs examining the association between tocilizumab treatment and clinical outcomes in COVID-19 patients (Figure 3). We recorded the meta-analysis under the Preferred Reporting Items for Systematic Reviews and Meta-Analyses (PRISMA) guidelines.

### Search strategy

Three review authors (Jingwen Peng, Weida Liu, and Huan Mei) systematically searched PubMed and MedRxiv for all RCTs as of June 1, 2021, to assess the benefits or harms of tocilizumab to treat patients with COVID-19 pneumonia (Figure 3). We additionally reviewed the references for included articles and previous systematic reviews. We compared included items and resolved disagreements.

### RCTs selection

The selected RCTs included participants with suspected or confirmed SARS-CoV-2 infection randomly assigned to receive tocilizumab, only standard of care or best supportive care, or a placebo together with the standard of care. We included all RCTs regardless of the tocilizumab dose (i.e., 400 mg-800 mg, 1.6 mg/kg and weekly continued with 0.8 mg/kg dose regimen or 8 mg/kg maximum 800 mg) or health care setting. We excluded retrospective studies, case reports, and the RCTs designed to prevent the occurrence of COVID-19.

### Data extraction

We carefully extracted the relevant information for all RCTs: baseline characteristics of the patients, trial design characteristics (Trial registration, blinding, and randomization procedure), description of the experimental and control groups, tocilizumab dose, and trial location. Data on outcomes (Jingwen Peng and Huan Mei) and features (Jingwen Peng and Weida Liu) were extracted independently by two reviewers.

### Outcomes

The outcomes were:

1. All-cause mortality 28 days or 30 days.
2. In-patient discharge rate.
3. The number of patients experiencing serious adverse events and adverse events.
4. The number of secondary infections.
5. The number of patients’ intubation or death.

### Risk of bias assessment

Two investigators (Jingwen Peng and Weida Liu) independently assessed the risk of bias for clinical outcomes in all trials using the internationally recognized tool (Revised Cochrane risk of bias tool for randomized trials, RoB 2.0). All authors accounted for any discrepancies in the investigator’s quality assessment and discussed until everyone reached a consensus.

### Statistical analyses

We performed the meta-analysis to assess the treatment effects using risk ratio (RR) and corresponding 95% confidence intervals (CI). We analyzed outcomes with available data (all-cause mortality, in-patient discharge rate, number of patients experiencing serious adverse events and adverse events, number of secondary infections, and patient intubation or death). We use DerSimonian and Laird methods to pool data from the meta-analysis with the random-effects model and the fixed-effects model of the Mantel-Haenszel method. We used Begg/Egger test and visually on a funnel plot to assess the meta-analysis and examine publication bias. We have not summarized treatment effects for clinical improvement or deterioration, length of hospital stay, and the number of mechanical ventilation due to inconsistent definitions of these outcomes and insufficient reporting of pertinent details. All analyses were carried out using RevMan version 5.4.1.

### Data availability

The datasets generated during and analyzed during the current study are available from the corresponding author on reasonable request.

## Supplementary Material

Supplementary Figures

Supplementary Table 1
