# Supplementary material for: Association between tocilizumab treatment and clinical outcomes of COVID-19 patients: a systematic review and meta-analysis
Source: Aging (Albany NY). 2022 Jan 17;14(2):557–71. doi: 10.18632/aging.203834 (PMC8833131; doi:10.18632/aging.203834)
Supplement: Supplementary Figures [file aging-14-203834-s001.pdf]

SUPPLEMENTARY FIGURES

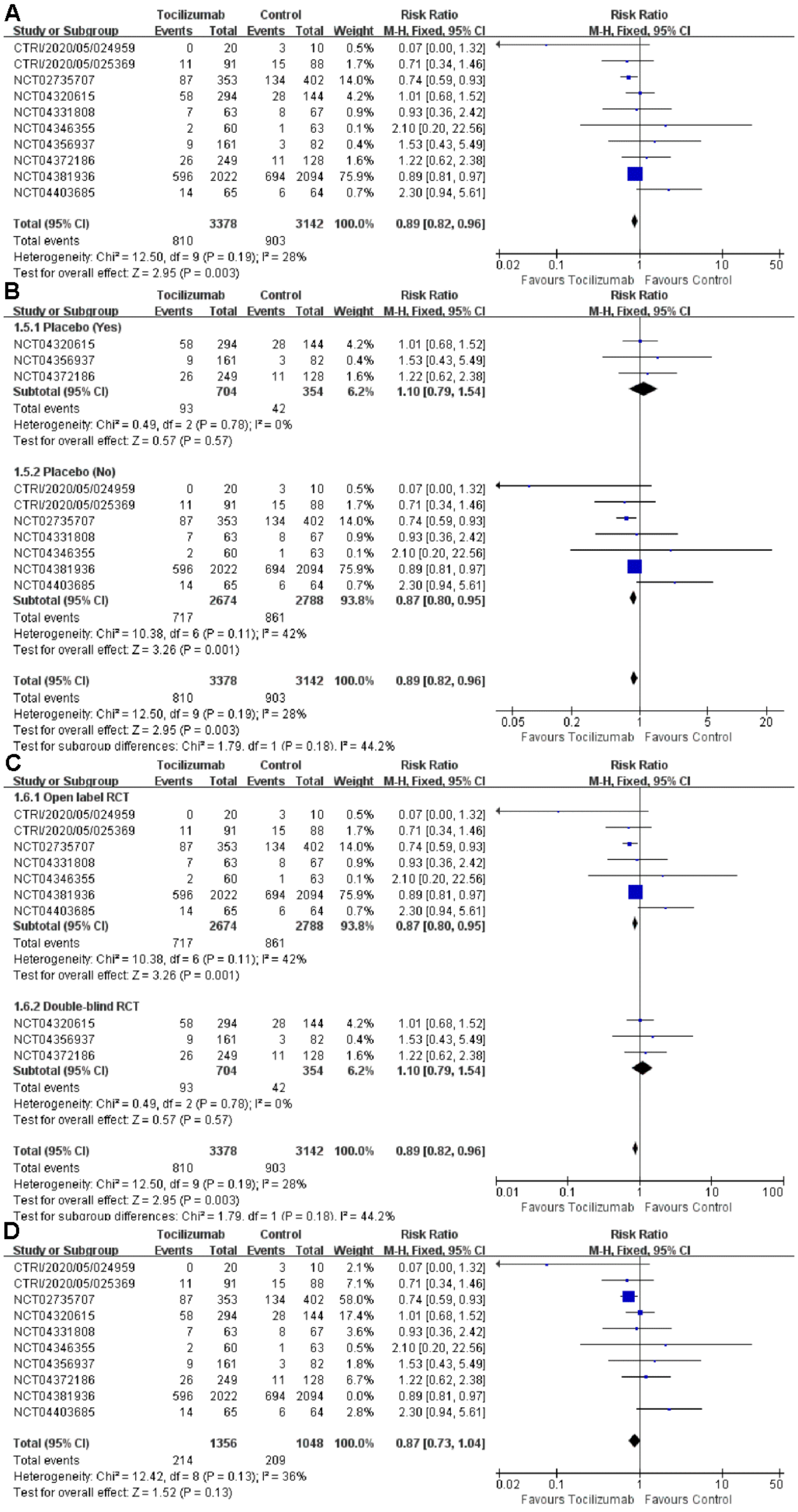

Supplementary Figure 1. Association of tocilizumab with all-cause mortality (**A**) all RCTs (**B**) study type (**C**) type of control (**D**) Forest plot without the RECOVERY trial.

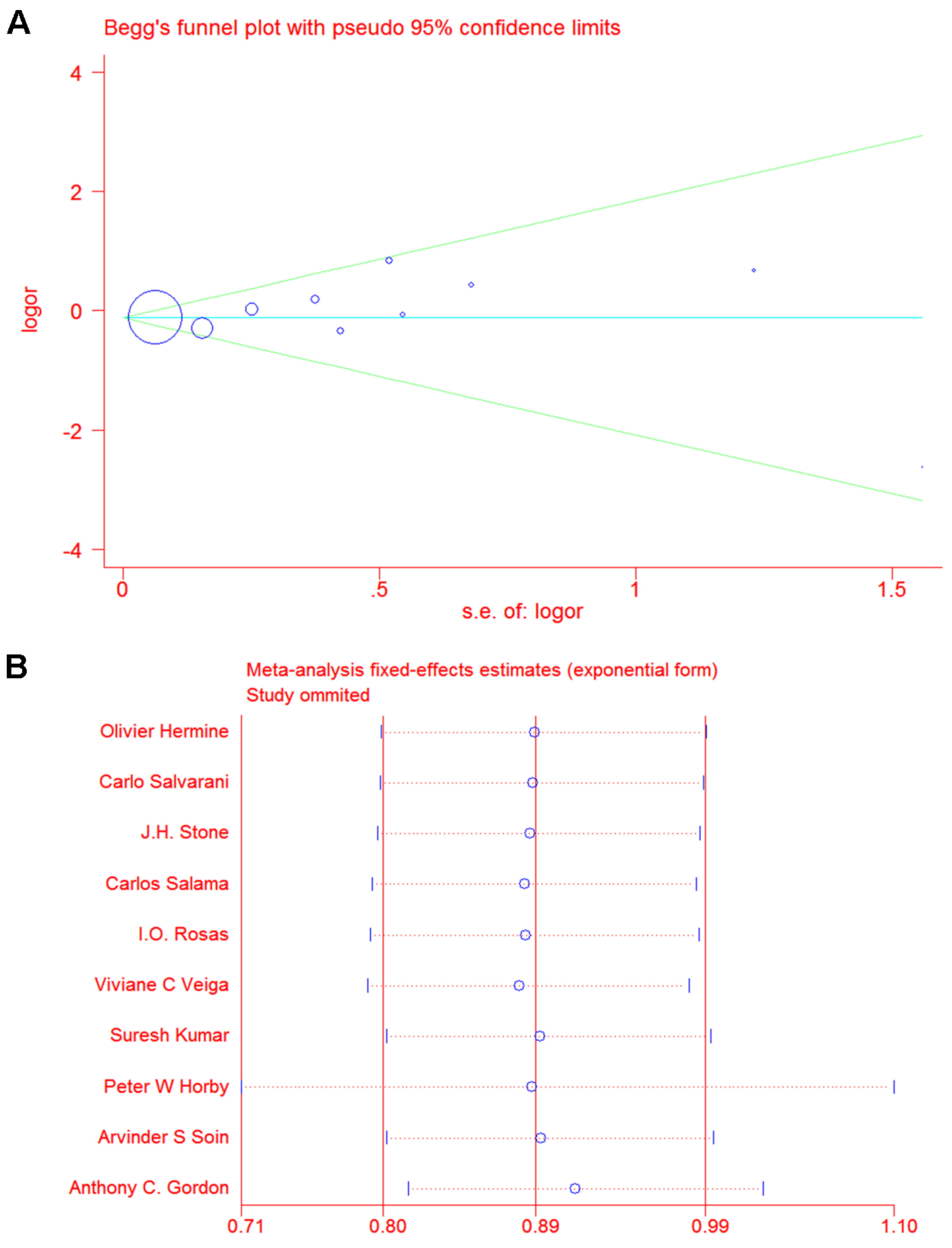

Supplementary Figure 2. Association of tocilizumab with all-cause mortality (**A**) funnel plot to assess the publication bias of the meta-analysis (**B**) sensitivity analysis.

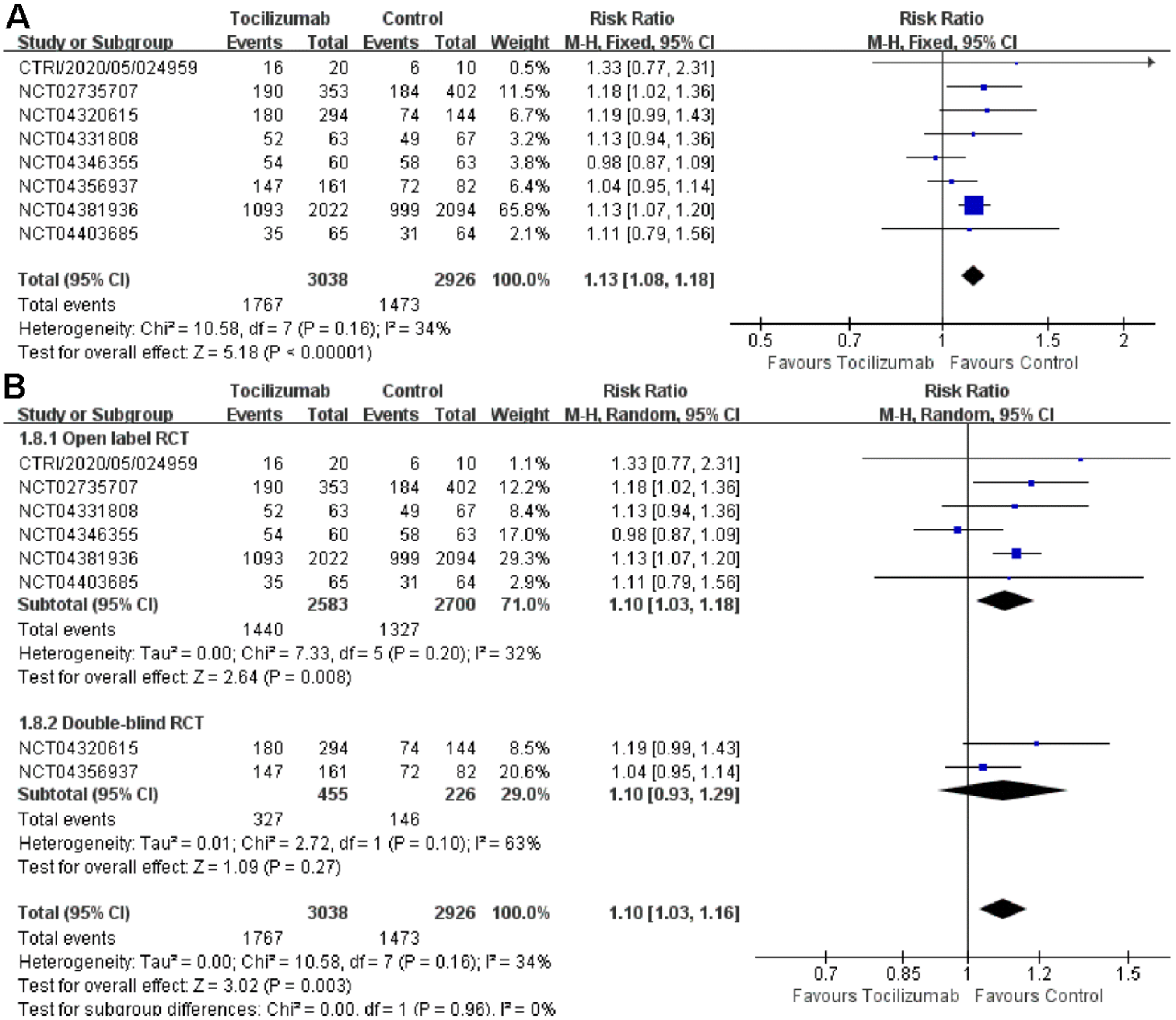

Supplementary Figure 3. Association of tocilizumab with discharge (**A**) all RCTs (**B**) study type.

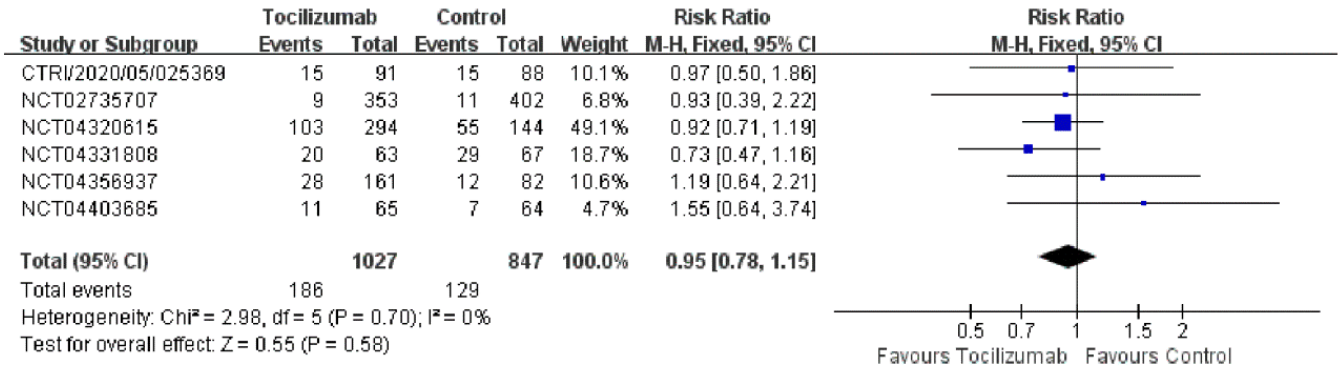

Supplementary Figure 4. Association of tocilizumab with patients of serious adverse events.

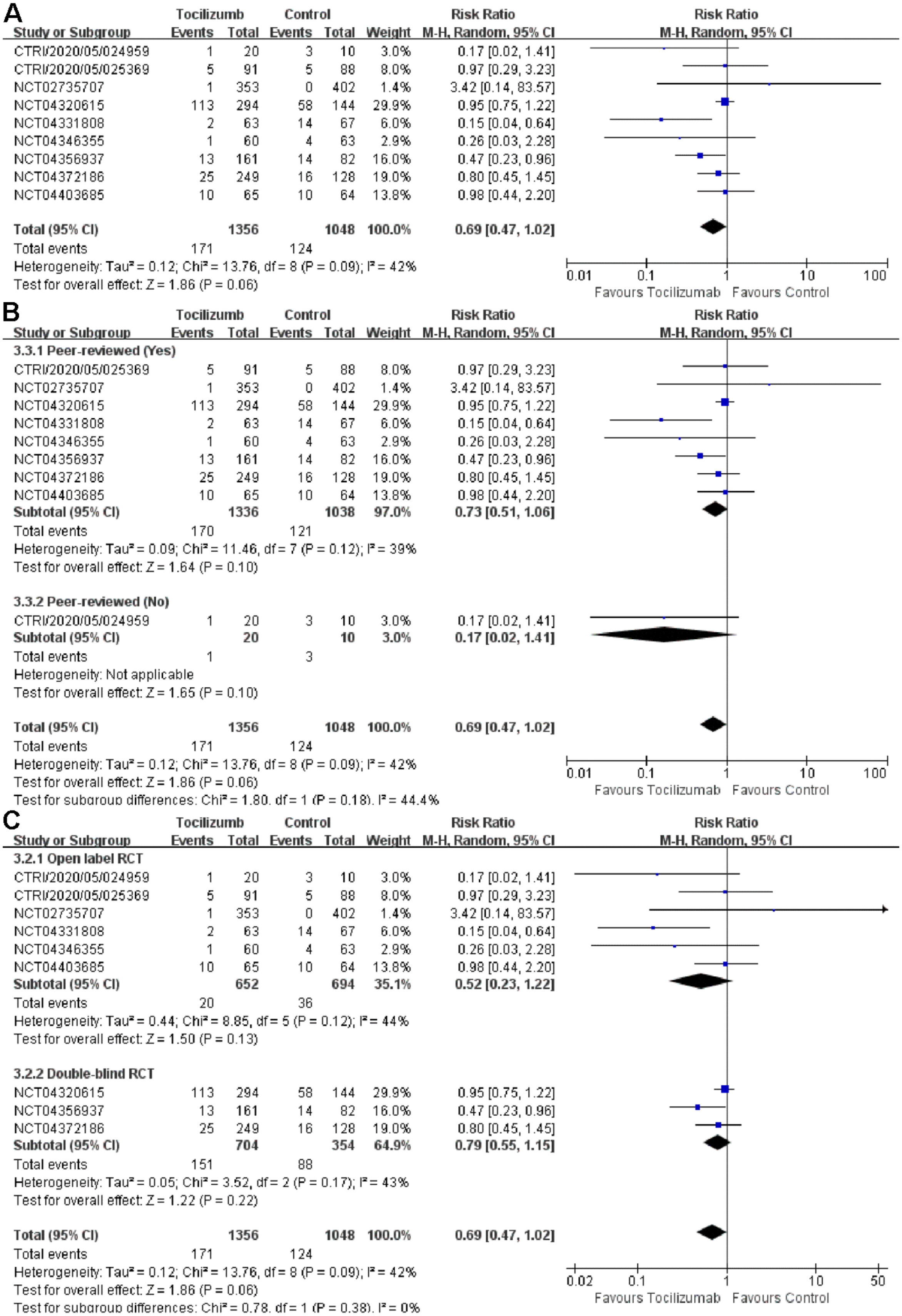

Supplementary Figure 5. Association of tocilizumab with secondary infections risk (**A**) all RCTs (**B**) peer-reviewed (**C**) type of control.
