# Supplementary material for: Association between tocilizumab treatment and clinical outcomes of COVID-19 patients: a systematic review and meta-analysis
Source: Aging (Albany NY). 2022 Jan 17;14(2):557–71. doi: 10.18632/aging.203834 (PMC8833131; doi:10.18632/aging.203834)
Supplement: Supplementary Table 1 [file aging-14-203834-s002.pdf]

SUPPLEMENTARY TABLE

Supplementary Table 1. Patient baseline characteristics of 8 RCTs in the meta-analysis.

Author		Olivier Hermine	Carlo Salvarani	J.H. Stone	Carlos Salama	I.O. Rosas	Viviane C Veiga	Suresh Kumar	Arvinder S Soin	Anthony C. Gordon	Peter W Horby
Trial registration		NCT 04331808	NCT 04346355	NCT 04356937	NCT 04372186	NCT 04320615	NCT 04403685	CTRI/2020/05/024959	CTRI/2020/05/025369	NCT 02735707	NCT 04381936
Treatment since symptom onset d	Tocilizumab	10 (7-13) (n=62)	7.0 (4.0-11.0)	9.0 (6.0–13.0)		12.1±6.6 (n=291)	10.0±3.1				9 (7-13)
	Control	10 (8-13) (n=66)	8.0 (6.0-11.0)	10.0 (7.0–13.0)		11.4±6.9 (n=143)	9.5±3.0				10 (7-14)
Age	Tocilizumab	64.0 (57.1-74.3)	61.5 (51.5-73.5)	61.6 (46.4–69.7)	56.0±14.3	60.9±14.6	57.4±15.7	49.55±12.49	56 (47–63)	61.5±12.5	63.3 ±13.7
	Control	63.3 (57.1-72.3)	60.0 (54.0-69.0)	56.5 (44.7–67.8)	55.6±14.9	60.6±13.7	57.5±13.5	48.30±14.62	54 (43–63)	61.1±12.8	63.9 ±13.6
Gender (Male/Female)	Tocilizumab	44/19	40/20	96/65	150/99	205/89	44/21	19/1	76/15	261/92	1335/687
	Control	44/23	37/29	45/37	73/55	101/43	44/20	7/3	76/12	283/119	1437/657
Hypertension	Tocilizumab		27	80		178	30		36		
	Control		29	38		94	34		34		
Diabetes	Tocilizumab	20(n=61)	10	45		105	22		31		569
	Control	23(n=67)	9	30		62	20		43		600
Cardiac disease	Tocilizumab	20(n=61)		17		88	4		15		435
	Control	20(n=67)		7		35	3		12		497
Pulmonary disease	Tocilizumab	3(n=61)	2	15		49	2		1		473
	Control	3(n=67)	2	7		22	2		2		484
Chronic kidney disease	Tocilizumab	5(n=61)		29			5		4		118
	Control	13(n=67)		13			1		4		99
Cancer	Tocilizumab	4(n=61)		22			5				
	Control	5(n=67)		8			5				
Asthma	Tocilizumab	5(n=61)		15			4				
	Control	3(n=67)		7			1				
Liver disease	Tocilizumab					6					14
	Control					2					10
Body mass index(BMI)	Tocilizumab	27.9 (23.3-30.8) (n=46)		29.9 (26.0–34.2)	32.0±7.9						
	Control	27.4 (24.5-31.3) (n=46)		30.2 (25.7–33.8)	33.1±7.2						
Obesity (BMI≥ 30)	Tocilizumab		16	80		63	15				
	Control		22	42		27	16				
